# Research on High-Stability Composite Control Methods for Telescope Pointing Systems under Multiple Disturbances

**DOI:** 10.3390/s24092907

**Published:** 2024-05-02

**Authors:** Rui Zhang, Kai Zhao, Sijun Fang, Wentong Fan, Hongwen Hai, Jian Luo, Bohong Li, Qicheng Sun, Jie Song, Yong Yan

**Affiliations:** MOE Key Laboratory of TianQin Mission, TianQin Research Center for Gravitational Physics & School of Physics and Astronomy, Frontiers Science Center for TianQin, Gravitational Wave Research Center of CNSA, Sun Yat-sen University (Zhuhai Campus), Zhuhai 519082, China; zhangr283@mail2.sysu.edu.cn (R.Z.);

**Keywords:** space gravitational wave, breathing angle variation, telescope pointing mechanism, *H_∞_* controller, HODOB, pointing stability

## Abstract

During the operation of space gravitational wave detectors, the constellation configuration formed by three satellites gradually deviates from the ideal 60° angle due to the periodic variations in orbits. To ensure the stability of inter-satellite laser links, active compensation of the breathing angle variation within the constellation plane is achieved by rotating the optical subassembly through the telescope pointing mechanism. This paper proposes a high-performance robust composite control method designed to enhance the robust stability, disturbance rejection, and tracking performance of the telescope pointing system. Specifically, based on the dynamic model of the telescope pointing mechanism and the disturbance noise model, an *H_∞_* controller has been designed to ensure system stability and disturbance rejection capabilities. Meanwhile, employing the method of an *H_∞_* norm optimized disturbance observer (HODOB) enhances the nonlinear friction rejection ability of the telescope pointing system. The simulation results indicate that, compared to the traditional disturbance observer (DOB) design, utilizing the HODOB method can enhance the tracking accuracy and pointing stability of the telescope pointing system by an order of magnitude. Furthermore, the proposed composite control method improves the overall system performance, ensuring that the stability of the telescope pointing system meets the 10 nrad/Hz^1/2^ @0.1 mHz~1 Hz requirement specified for the TianQin mission.

## 1. Introduction

In 2015, the LIGO (Laser Interferometer gravitational-wave Observation) team detected gravitational waves for the first time, ushering in a new era of exploring gravitational waves in the universe [1]. The LISA (Laser Interferometer Space Antenna) mission proposed jointly by ESA and NASA consists of three spacecraft deployed on a heliocentric orbit, aiming to detect space-based gravitational wave sources in the frequency range of 0.1 mHz to 1 Hz [2]. Similar to LISA, the TianQin project plans to deploy three spacecraft in Earth’s orbit at an altitude of approximately 100,000 km, forming an equilateral triangle constellation with an arm length of about 170,000 km. By utilizing a telescope system to receive and transmit laser beams, a laser link is established between each pair of spacecraft. The detection of space gravitational waves is achieved by measuring the variation in the distance between two test masses [3].

During gravitational wave detection, the constellation formed by three spacecraft may gradually deviate from the ideal equilateral triangle shape over time due to the influence of celestial gravitational perturbations and initial orbital deviations. This deviation is known as the breathing angle variation. After optimizing the constellation configuration for stability, the TianQin constellation can control the breathing angle variation within a range of ±0.1° during each three-month observation window [4]. However, the variation in the breathing angle remains significantly larger than the far-field beam width of the telescope (about 5 μrad) [5], leading to a misalignment of the lines of sight for the two spacecraft, and thereby affecting the normal detection of gravitational waves. Therefore, to ensure the detection of gravitational waves, the TianQin mission specifies a pointing stability requirement of 10 nrad/Hz^1/2^ in the frequency range of 0.1 mHz to 1 Hz [6] and utilizes the pointing mechanism to actively compensate for the breathing angle variation.

Currently, in space gravitational wave detection, there are two pointing schemes used to compensate for the breathing angle variation within the TianQin constellation. The first scheme is the whole telescope pointing scheme, which involves using the telescope pointing mechanism to rotate the entire mobile optical subassembly (MOSA, consisting of a telescope, optical bench, gravitational reference sensor, telescope pointing mechanism, supporting structures, etc.) to compensate for angular changes. The second scheme is the in-field pointing scheme [7], which entails designing a rotatable mirror within the optical system of the telescope to compensate for angular changes. However, the second scheme increases the complexity of the telescope’s optical design and introduces additional stray light [8], thereby affecting the precision of distance measurement. Therefore, using the whole telescope pointing to compensate for the breathing angle variation becomes a feasible solution.

In the whole telescope pointing scheme, the stability of the telescope pointing mechanism is compromised due to the influence of unfavorable conditions, such as model uncertainties, disturbance torque noise, and nonlinear friction. Therefore, it is essential to mitigate the impact of these adverse factors to improve the precision and stability of the telescope pointing system. Currently, for the telescope’s high-precision pointing control methods, Thomas et al. [9] have employed a combination of robust loop shaping and disturbance observer methods to ensure robust stability across the entire operational range. This approach enhances the performance of precision satellite systems and reduces the servo error by a factor of 3.8. Wang et al. [10], using a stacked recursive neural network adaptive controller, addressed the issue of insufficient control precision, achieving precise pointing requirements at the nanoradian level between satellites and telescopes. Deng et al. [11] designed a frequency-divided controller that coordinates the spacecraft attitude control loop and the telescope attitude control loop, improving the overall performance and pointing stability of the system. Cao et al. [12] proposed a closed-loop control method for a fine stabilization system based on dual-port adaptive internal model control, enabling the disturbance compensation of the space telescope’s fine stabilization system with lower steady-state error and a broader frequency range. In addition, for robust control in space applications, Serhii Khoroshylov et al. [13] proposed an attitude controller of a mini-SSAR with a deployable reflector antenna, which ensures robustness against unmodeled dynamics and frequency variations. Zhao et al. [14] proposed a layered sliding mode fault-tolerant tracking control method based on a zero-sum game, which improved the response speed and robustness of the system. Wu et al. [15] proposed a low-computation two-level triggering adaptive control strategy to achieve accurate trajectory tracking and maintain the boundedness of closed-loop signals.

However, the aforementioned studies mainly focus on the coordinated control between spacecraft attitude and telescope pointing, with relatively simple disturbance noise. This may lead to a decline in control performance in the presence of multiple disturbances, making it challenging for traditional control methods to ensure high-precision pointing requirements. In terms of robust tracking control, the provided controller is not specifically designed for multiple disturbances, and thus, such control methods may be insufficient in suppressing these disturbances. To improve the system’s disturbance rejection capability, tracking accuracy, and pointing stability, a composite tracking control method that guarantees the system’s robust stability and dynamics while attenuating and suppressing multi-frequency disturbances has been proposed. This method has already been applied in several engineering fields [16,17].

In this paper, the problem of trajectory tracking in a telescope pointing system with ultra-high precision and ultra-high pointing stability under multiple disturbances is investigated. A composite tracking control method is designed by introducing an *H*_∞_ controller in the outer loop of the control system and integrating a robust disturbance observer in the inner loop. This method aims to improve the disturbance rejection capability of the system and ensure that the tracking accuracy and pointing stability of the telescope pointing system meet the requirements of gravitational wave detection tasks. The main contributions of this paper are summarized as follows:

1. Disturbance analysis: Based on the mechanism research, this paper systematically analyzes the multiple disturbance noises of the telescope pointing system and establishes a specific disturbance mathematical model. This lays a solid foundation for suppressing multiple disturbances and improving system pointing stability.
2. Controller design: Based on the dynamic model of the telescope pointing mechanism and the multiple disturbance model, this paper designs a composite control controller. By designing an *H*_∞_ controller, stability is ensured while suppressing external disturbance noise. To relax the constraints of the feedback controller and enhance the anti-interference capability of the telescope pointing system, an HODOB method is proposed. By combining the *H*_∞_ controller and HODOB, it not only suppresses and attenuates multiple interferences but also achieves satisfactory tracking accuracy and pointing stability level, compensating for the respiratory angle changes in gravitational wave detection.

The paper is organized as follows: Section 2 establishes the dynamic models of the telescope pointing mechanism and analyzes the models of the primary disturbance noises. Section 3 is dedicated to presenting the primary contributions, where a composite control scheme is proposed. In Section 4, the simulation results are provided to demonstrate the effectiveness of the proposed scheme, and Section 5 concludes the paper.

## 2. Pointing Strategy and Model Establishment

### 2.1. Pointing Scheme and Maneuvering Requirements

**Figure 1:**
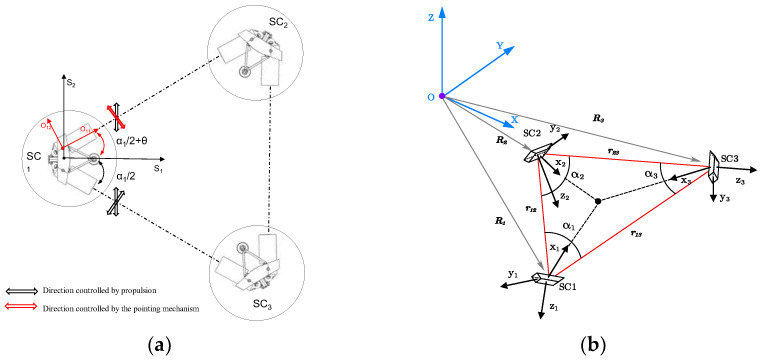
Pointing scheme for breathing angle compensation. (**a**) Pointing scheme. (**b**) Coordinate system relationship.

In the scientific mode, to ensure the precision of space gravitational wave detection, fewer movable components inside the spacecraft are preferred. Each spacecraft in the TianQin mission consists of two MOSAs. To enhance the detection accuracy, we propose a single MOSA pointing scheme, as illustrated in Figure 1a. One of the MOSAs is adjusted through the telescope pointing mechanism to compensate for the breathing angle variation *θ* within the TianQin constellation plane. Simultaneously, a pointing adjustment device is also installed on the other MOSA as a backup mechanism.

Additionally, the out-of-plane angle variations in the TianQin constellation are compensated for by adjusting the spacecraft’s attitude using micro-propulsion devices; however, this aspect is not considered in this paper. Given the complex coupling relationship between the spacecraft and the telescope, a decoupling analysis was conducted, treating them separately. Through the design of a single-input–single-output (SISO) controller, control of the telescope pointing mechanism’s single degree of freedom is achieved [18].

The spacecraft reference frame (O_i_-x_i_y_i_z_i_) and the inertial reference frame (O-XYZ) was established, as shown in Figure 1b. The maneuvering requirements for telescope pointing were computed using satellite orbit data [19].

The arm length *r_ij_* between spacecraft *i* and *j*, where *i*, *j* ∈ (1,2,3), can be expressed in an inertial reference frame.

(1) $$r_{ij}=\left\|r_{ij}\right\|=\left\|R_{i}-R_{j}\right\|,$$

where ***R****_i_* represents the position of the spacecraft SCi in the inertial reference frame. Define the unit vector as ***n****_ij_* = ***r****_ij_*/|***r****_ij_*|. The angle *α_i_* between the sight lines of the telescopes in the spacecraft SCi can be expressed as

(2) $$\alpha_{i}=\arccos(n_{ij}\cdot n_{ik})=\arccos(\frac{r_{ij}\cdot r_{ik}}{\left\|r_{ij}\right\|\times \left\|r_{ik}\right\|}).$$

**Figure 2:**
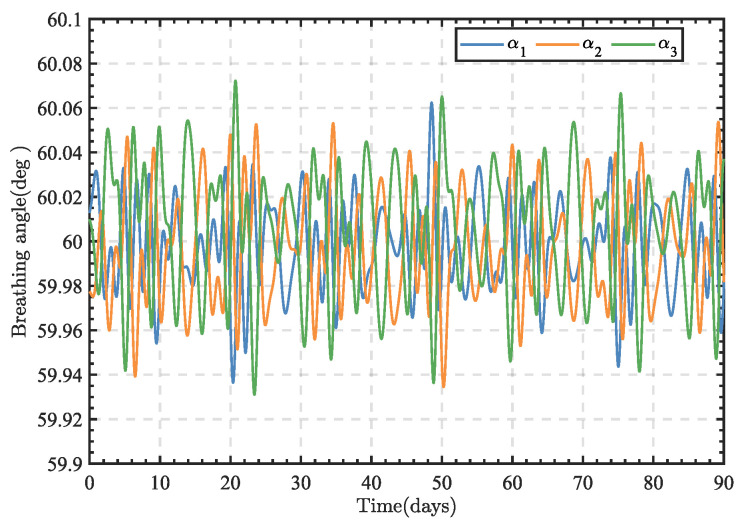
Time variations in the three breathing angles α_1,2,3_ of the constellation.

Substituting the changes in the satellite orbits in the inertial coordinate system equation into Equation (2), the trajectory variations in the breathing angle among the three spacecraft over three months can be obtained, as illustrated in Figure 2. It can be observed in the figure that the breathing angle variation in the TianQin constellation did not exceed ±0.1° over the three months.

### 2.2. Dynamic Model

#### 2.2.1. Structural Model

**Figure 3:**
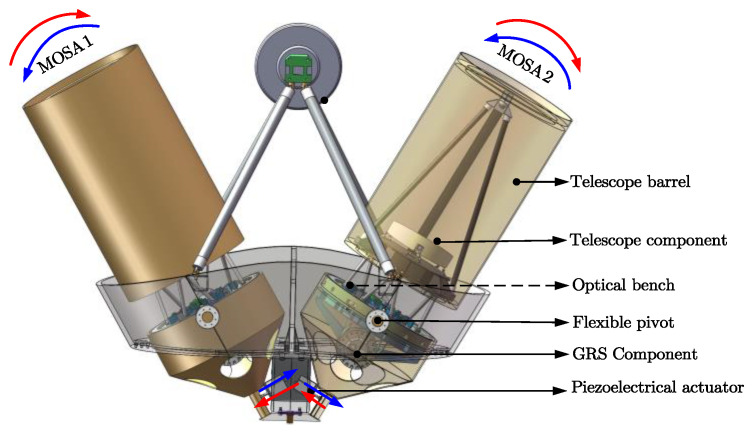
Three-dimensional model of the telescope pointing mechanism.

To achieve ultra-high stability tracking compensation for the TianQin breathing angle variation, stringent requirements were imposed on the structural design of the telescope pointing mechanism. To ensure the stability of the pointing mechanism, a flexible bearing was chosen for the rotation axis, and high-precision piezoelectric actuators are used to drive the MOSA rotation in a direct-drive manner. Figure 3 provides the preliminary three-dimensional model of the telescope pointing mechanism in the TianQin mission.

#### 2.2.2. Coordinate System Definition

**Figure 4:**
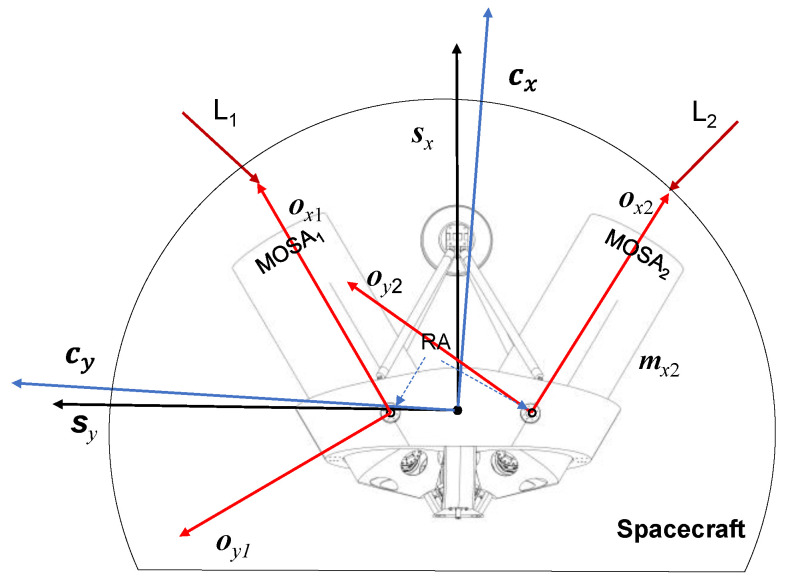
Reference systems.

To facilitate the establishment of the dynamic model of the telescope pointing mechanism, we established the following coordinate systems as reference frames, and the spatial relationships among these coordinate systems are illustrated in Figure 4.

- The Constellation Reference Frame, CRF = {O_C_,***c****_x_*,***c****_y_*,***c****_z_*}, with the origin located at the center of mass of the spacecraft, where ***c****_z_* is perpendicular to the plane formed by the incident laser vectors L2 and L3, ***c****_x_* is the bisector of the angles defined by the two incident lasers, and ***c****_y_* satisfies the right-hand rule;
- Two Optical Reference Frames, ORF = {O_o*j*_,***o****_xj_*,***o****_yj_*,***o****_zj_*}. The coordinate origin is defined on the rotation axis of the optical subassembly, and the center of mass of the optical subassembly is also located at this point. The ***o****_xj_* axis is oriented along the symmetry axis of the optical subassembly and points toward the laser emission direction. The ***o****_yj_* axis is perpendicular to the ***o****_xj_* axis and points in the opposite direction of the angle between the two optical subassemblies. The ***o****_zj_* axis coincides with the rotation axis of the optical subassembly, following the right-hand rule;
- The Spacecraft Reference Frame, SRF = {O_S_,***s****_x_*,***s****_y_*,***s****_z_*}. The coordinate origin is defined at the center of mass of the entire spacecraft. The ***s****_x_* axis direction is between the two telescopes, pointing in a direction that forms a 30° angle with the optical reference frames’ ***o****_xj_* axis direction. The ***s****_y_* axis direction is perpendicular to the ***s****_x_* axis direction and coincides with the planes of the two optical components. The ***s****_z_* axis direction is perpendicular to both the planes of the optical subassembly and follows the right-hand rule.

#### 2.2.3. Mechanism Dynamics Model

The angular acceleration of the telescope pointing mechanism can be obtained according to the angular momentum theorem:

(3) $${\dot{H}}_{O}=J_{t}{\ddot{\theta }}_{O},$$

where ***θ***_O_ is the rotation angle of the pointing mechanism, and *J_t_* is the moment of inertia. According to the conservation of angular momentum, *Ḣ*_O_ = external torque—internal torque. Consider using a preliminary second-order model to describe the dynamics of the telescope pointing mechanism:

(4) $$\begin{array}{l} {\ddot{\theta }}_{O}=-2\omega_{N}\xi {\dot{\theta }}_{O}-\omega_{N}^{2}\theta_{O}+J_{t}^{-1}(M_{T}^{O}+D_{T}^{O})-J_{t}^{-1}(M_{T}^{f}+M_{T}^{\mathrm{SO}}+M_{T}^{\mathrm{EO}})\\ \omega_{N}=\sqrt{\frac{K}{J_{t}}},\;\xi =\frac{B}{2\sqrt{KJ_{t}}} \end{array},$$

where *ω*_N_ is the natural frequency, *ξ* is the damping coefficient, and K, B are the stiffness and damping, respectively. $M_{T}^{O}$ is the driving torque generated by the piezoelectric actuator, $D_{T}^{O}$ is the external disturbance torque acting on the pointing mechanism, primarily due to the gravity gradient torque, $M_{T}^{f}$ is the internal friction torque of the telescope pointing mechanism, **$M_{T}^{SO}$** is the spacecraft reaction torque on the telescope pointing mechanism, and **$M_{T}^{EO}$** is the reaction torque of the test mass (TM) on the telescope pointing mechanism. Since the TM is located inside the telescope pointing mechanism, the magnitude of the reaction torque on the mechanism is equal to the torque applied to the TM.

#### 2.2.4. Actuator Model

**Figure 5:**
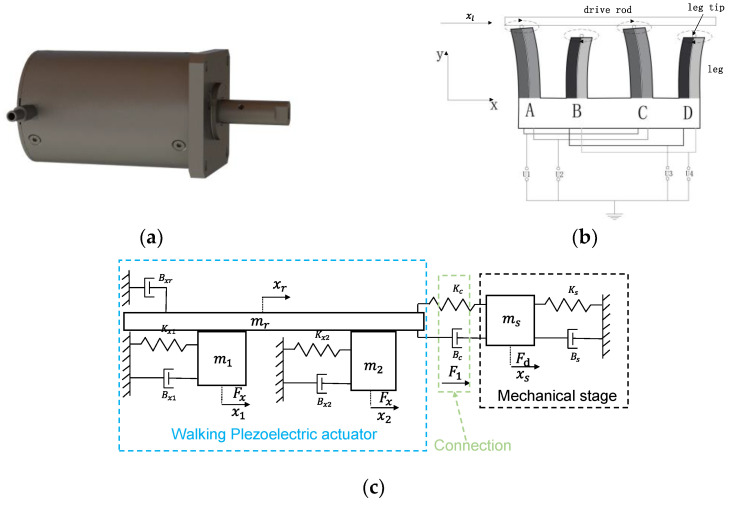
Walking piezo actuator. (**a**) Three-dimensional model of the piezoelectric actuator. (**b**) Schematic representation of the working principle of the piezoelectric actuator. (**c**) Equivalent model of the piezoelectric actuator.

For the telescope pointing mechanism, the selection of its drive device has a significant impact on the performance of the mechanism. According to the requirements of gravitational wave detection, the following restrictions are imposed on the drive device: (1) no electromagnetic noise is allowed, and (2) requirements for driving force, stroke, and precision must be met. Based on the above analysis, we chose a walking piezo actuator as the drive device for the pointing mechanism, as shown in Figure 5a. Figure 5b illustrates the working principle of the walking piezo actuator, where the motion displacement is formed by the alternating interaction of two pairs of piezoelectric legs with the drive rod.

The expression for the driving torque of a telescope pointing system is as follows:

(5) $${Μ}_{T}^{O}=F_{d}\times R,$$

where *R* represents the moment arm, which refers to the distance between the actuator’s point of action and the axis of rotation of the telescope pointing mechanism, and *F*_d_ represents the driving force generated by the actuator. Figure 5c illustrates the equivalent model of the piezoelectric actuator, from which a mathematical model can be derived. Due to space limitations, this paper does not provide the specific derivation process [20].

(6) $$\left\{\begin{array}{l} F_{x}=K_{x}x_{p}=K_{x}c_{px}\Delta U\\ F_{1}-2F_{x}=M{\ddot{x}}_{r}+B{\dot{x}}_{r}+Kx_{r}\\ F_{1}=B_{c}\left({\dot{x}}_{r}-{\dot{x}}_{s}\right)+K_{c}\left(x_{r}-x_{s}\right)\\ F_{1}+F_{d}=M_{s}{\ddot{x}}_{s}+B_{s}{\dot{x}}_{s}+K_{s}x_{s} \end{array}\right.,$$

where *F_x_* is the piezoelectric electromotive force, *F*_1_ represents the external force applied to the piezoelectric actuator from the connection part, *K_x_* is the equivalent spring coefficient, *c*_px_ is the voltage coefficient, ∆*U* is the voltage difference, M, B, and K are the equivalent mass, damping, and stiffness in the horizontal direction of the piezoelectric actuator, K_c_ and B_c_ are the equivalent damping and stiffness of the connecting components, and M*_s_*, B*_s_,* and K*_s_* are the equivalent mass, damping, and stiffness, respectively, of the mechanical stage in the horizontal direction.

### 2.3. Disturbance Analysis and Descriptions

#### 2.3.1. Reaction Torque from the Spacecraft on the Telescope Pointing Mechanism

When the spacecraft adjusts its attitude in orbit, the forces and torques applied by the micro-thrusters will have a certain impact on the telescope pointing mechanism. The influencing torque **$M_{T}^{SO}$** can be expressed as:

(7) $$M_{T}^{\mathrm{SO}}=T_{S}^{Oj}J_{t}{\dot{\omega }}_{\mathrm{SI}},$$

where ***ω***_SI_ represents the spacecraft’s inertial angular velocity in the SRF coordinate system, which can be obtained through dynamic modeling of the spacecraft. $T_{S}^{Oj}$ is the transformation matrix from the spacecraft to the pointing mechanism, and according to coordinate definitions, the matrix is a rotation matrix about the ***o****_zj_* axis.

The attitude dynamics equations of a spacecraft:

(8) $$\begin{array}{l} {\dot{\omega }}_{\mathrm{SI}}=-J_{S}^{-1}\omega_{\mathrm{SI}}\times J_{S}\omega_{\mathrm{SI}}+J_{S}^{-1}({Μ}_{T}^{S}+D_{T}^{S}+D_{⊙press}^{S})\\ \;\;-J_{S}^{-1}\sum_{j=1,2}(T_{Oj}^{S}J_{t}{\ddot{\theta }}_{O}^{j}+T_{Oj}^{S}M_{Ej}^{Oj}+b_{j}^{S}\times T_{Oj}^{S}F_{Ej}^{Oj}) \end{array},$$

where ***J***_S_ is the spacecraft’s inertia matrix, $M_{T}^{S}$ is the torque provided by thrusters, $D_{T}^{S}$ is the torque noise from thrusters, $D_{⊙press}^{s}$ is the torque noise from solar radiation pressure, $M_{Ej}^{Oj}$ represents the torque generated on the TM, $F_{Ej}^{Oj}$ represents the force generated on the TM, $b_{j}^{S}$ denotes the position of the center of mass of the TM relative to the spacecraft, $T_{Oj}^{S}$ is the transformation matrix between the telescope pointing mechanism and the spacecraft.

**Figure 6:**
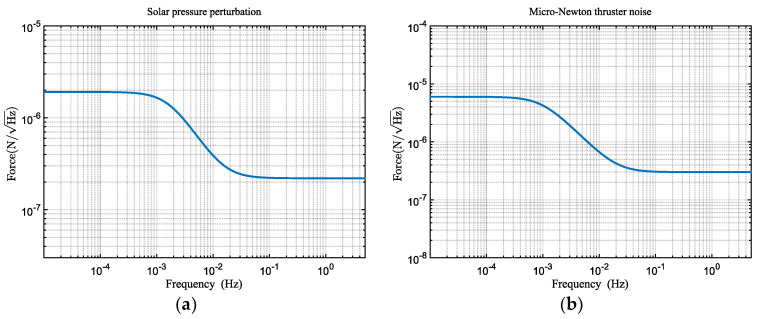
The noise curve. (**a**) Solar radiation pressure noise. (**b**) Micro-propulsion thruster thrust noise.

The solar radiation pressure torque on the spacecraft refers to the torque generated by the pressure exerted by sunlight on the spacecraft’s surface. This torque induces deviations from the pure gravitational orbital motion of the spacecraft. The solar radiation pressure torque can be expressed as:

(9) $$D_{⊙press}^{S}=\sum_{i=1}^{n}l\times F_{⊙press},$$

where $F_{⊙press}$ is the solar radiation pressure, and *l* is the distance between the center of pressure and the center of mass of the illuminated surface. The solar radiation pressure torque model [21] used in this paper is depicted in Figure 6a. Additionally, in Equation (8), the spacecraft is subject to the thrust noise from micro-propulsion thrusters [21], and its noise model is illustrated in Figure 6b.

#### 2.3.2. Nonlinear Friction Torque

During the operation of the telescope pointing mechanism, the contact and movement between various components can result in the presence of frictional forces. Frictional torque induces speed fluctuations in the system, causing phenomena, such as dead-zone crawling, nonlinearity, and steady-state errors when the system operates at low speeds. Considering the trajectory of the breathing angle, the telescope pointing mechanism typically operates at low speeds. The presence of nonlinear friction can prevent the telescope pointing system from meeting high-precision pointing requirements. To enhance the control performance of the system, it is necessary to reduce or eliminate the impact of friction on speed stability.

The LuGre model can accurately and comprehensively describe the static friction characteristics and the presence of dynamic friction phenomena in the telescope pointing mechanism [22]. This model employs an elastic bristle u to simulate the structure of the contact surface, and the frictional torque can be represented as:

(10) $$\begin{array}{l} M_{T}^{f}=\lambda_{0}z+\lambda_{1}\dot{u}+\lambda_{2}v\\ \dot{u}=v-u|v|/g(v)\\ \lambda_{0}g(v)=F_{c}+\left(F_{s}-F_{c}\right)e^{-{\left(v/v_{s}\right)}^{2}} \end{array},$$

where *λ*_0_ is the stiffness coefficient, *λ*_1_ is the damping coefficient, *λ*_2_ is the velocity-dependent damping between the contact surfaces, *F*_c_ is the coulomb friction force, *F*_s_ is the static friction force, *v* is the relative velocity between the contact surfaces, vs. is the Stribeck velocity, *u* is the average deformation of the contact surface asperities, and *g(v)* is a function related to the variable *v*. According to Equation (10), the friction force is a disturbance related to the rotational speed of the pointing mechanism.

#### 2.3.3. Gravity Gradient Torque

Each small mass element within the MOSA experiences the gravitational force of the Earth. Due to the non-coincidence of the rotation center of the telescope pointing mechanism with the center of mass of the spacecraft, the resultant gravitational force acting on the entire MOSA does not pass through the center of mass of the spacecraft. The torque caused by the gravitational gradient is referred to as the gravity gradient torque. The presence of the gravity gradient torque can degrade the tracking precision and stability of the telescope pointing system. The torque model can be expressed as follows:

(11) $$D_{G}=\frac{3\mu }{{\left|r\right|}^{5}}r\times (J_{t}\cdot r),$$

where *μ* is the gravitational constant of the Earth, and ***r*** is the vector pointing from the center of the Earth to the center of the probe.

#### 2.3.4. Other Noise

**Figure 7:**
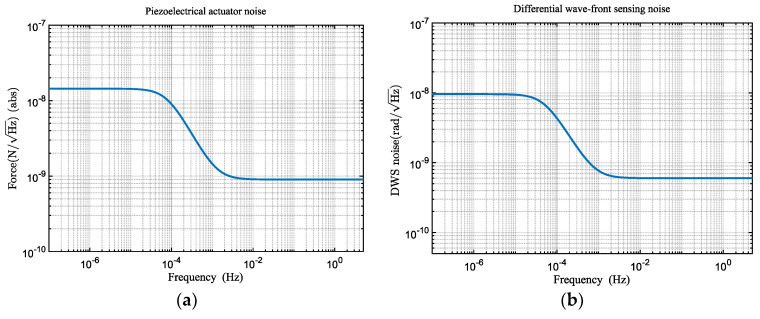
The noise curve. (**a**) Piezoelectric actuator drive noise. (**b**) DWS measurement noise.

In addition to the aforementioned primary disturbance noises, the telescope pointing system is also subject to other noise influences: (1) actuator drive noise, which is the torque noise generated by the piezoelectric actuators in the telescope pointing mechanism [11], as illustrated in Figure 7a; (2) sensor noise. To achieve angle measurements at the nanoradian level, we utilized differential wavefront sensing (DWS) for angular measurements [23], with the DWS signal serving as the feedback control signal for the telescope pointing system. Figure 7b presents the DWS measurement noise [21].

Based on the above analysis, the telescope pointing system is subject to a complex and numerous set of disturbances, and these disturbance parameters may change continuously with varying operational conditions. Consequently, relying on model parameter identification to eliminate or reduce the impact of multiple disturbances on the telescope pointing system may not be an optimal choice. Moreover, the disturbances affecting the system span different frequency bands, posing a significant challenge for controller design. Traditional anti-disturbance methods are primarily designed for single disturbance sources and are less effective in attenuating and suppressing disturbances across multiple frequency bands. In this paper, a high-performance controller is designed to ensure that the pointing stability of the system meets the requirements of the TianQin mission when compensating for changes in the breathing angle by the telescope pointing mechanism.

## 3. Design of Composite Control Method

**Figure 8:**
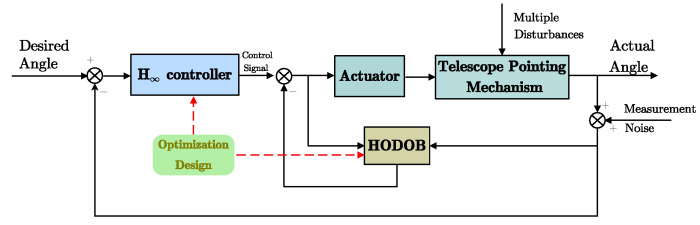
Composite control block scheme for telescope pointing system.

To ensure that the telescope pointing mechanism achieves high-precision and stable tracking under multiple disturbances, this section proposes a composite tracking control method. Firstly, an *H*_∞_ controller is designed to ensure the robust stability of the system, enhance disturbance rejection, and suppress sensor noise. Subsequently, a method based on *H*_∞_ norm optimization disturbance observer is designed to further suppress the disturbance noise of the telescope pointing mechanism, especially non-linear friction. Figure 8 illustrates the block diagram of the composite control method for telescope pointing.

### 3.1. H_∞_ Mixed Sensitivity Controller Design

**Figure 9:**
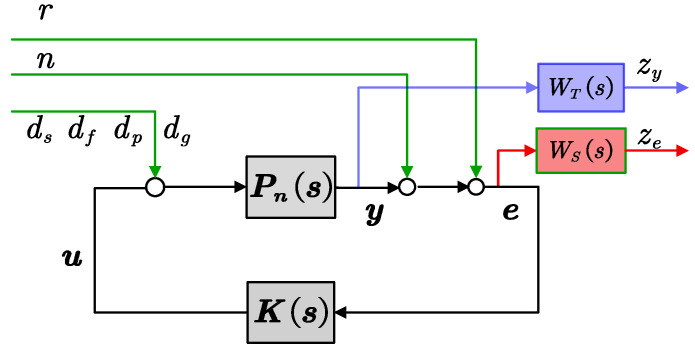
The structure of the *H*_∞_ mixed-sensitivity controller.

In general, system models often involve uncertainties, making the robust stability of the control system crucial. *H*_∞_ is a frequency-domain optimization method used for designing robust controllers to ensure stability and meet certain performance requirements in the presence of uncertainties and disturbances. In this paper, we have designed an *H*_∞_ mixed-sensitivity controller to address disturbance rejection and robust stability issues. The *H*_∞_ mixed-sensitivity control diagram is illustrated in Figure 9.

In Figure 9, *K*(*s*) is the designed feedback controller, *P*_n_(*s*) is the nominal model of the plant, *r* is the reference input model, *n* represents the sensor noise, and *d_s_*, *d_f_*, *d_g_*, and *d_p_* respectively denote the spacecraft reaction torque, nonlinear friction, gravity gradient torque, and piezoelectric actuator noise.

The sensitivity function *S*_0_(*s*) and complementary sensitivity function *T*_0_(*s*) of the system are defined as follows:

(12) $$S_{0}(s)=\frac{1}{1+P_{n}(s)K(s)},T_{0}(s)=\frac{P_{n}(s)K(s)}{1+P_{n}(s)K(s)}.$$

The sensitivity function *S*_0_(*s*) characterizes the transmission influence of the external input *r* on the control error *e* and the transmission influence of the measurement noise *n* on the measurement output *y.* The smaller the singular values of *S*_0_(*s*), the stronger the system’s tracking ability and the better the robust performance in suppressing input disturbances. To ensure small singular values of *S*_0_(*s*), it is required to minimize the ∞-norm of the sensitivity function, that is,

(13) $${\left\|W_{S0}(j\omega )\cdot S_{0}(j\omega )\right\|}_{\infty }<1,\;\forall \omega ,$$

where *W_S_*_0_(*s*) is a weighted function of *S*_0_(*s*), aiming to improve the tracking accuracy and attenuate low-frequency disturbance.

The complementary sensitivity function *T*_0_(*s*) characterizes the robust stability of the system in response to model multiplicative uncertainty. The smaller the singular values of *T*_0_(*s*), the better the robust performance. To ensure the stability of the telescope pointing system under multiplicative perturbation Δ(*s*), according to the small gain theorem [24], the robust stability condition of the closed-loop system is given by:

(14) $$\bar{\sigma }(\Delta (j\omega )T_{0}(j\omega ))<1,\;\forall \omega ,$$

where $\bar{\sigma }$ represents the maximum singular value. Assuming that the upper bound function of the magnitude frequency response for $\bar{\sigma }(∆\left(j\omega \right))<W_{T0}(j\omega )$ is *W_T_*_0_(*s*), the sufficient condition for robust stability is

(15) $${\left\|W_{T0}(s)T_{0}(s)\right\|}_{\infty }<1,$$

where *W_T_*_0_(*s*) represents the weighting function of *T*_0_(*s*), which represents the robustness against system uncertainty and measurement noise.

Due to the relationship *S*_0_(*s*) + *T*_0_(*s*) = 1 between *S*_0_(*s*) and *T*_0_(*s*), it is not possible for both of them to be small simultaneously. Therefore, there is a trade-off between the robustness and the performance of the system. In practical applications, a mixed sensitivity performance index is utilized for representation [25].

(16) $$\underset{K(s)}{\min}{\left\|\begin{array}{c} W_{S0}(s)S_{0}(s)\\ W_{T0}(s)T_{0}(s) \end{array}\right\|}_{\infty }<\gamma_{0},$$

where $0<\gamma_{0}<1$ represents the performance level of *H*_∞_. Based on the above analysis, the weighted function *W_S_*_0_(*s*) should possess low-pass characteristics to attenuate low-frequency disturbances. Meanwhile, the weighted function *W_T_*_0_(*s*) should possess high-pass characteristics to handle multiplicative disturbances and sensor noise.

The TianQin space gravitational wave detection frequency range is from 0.1 mHz to 1 Hz. In order not to affect the normal measurement of gravitational waves, the pointing control frequency of the telescope can be designed outside of the scientific frequency range. Specifically, the weighting function can be chosen as:

(17) $$W_{S0}(s)=\frac{0.3\;s+381.6}{\;s+3.816\times 10^{-4}},W_{T0}(s)=\frac{s+2.309\times 10^{2}}{\;0.001s+4.619\times 10^{2}}.$$

By utilizing the robust toolbox of Matlab, the *H*_∞_ controller can be computed as follows:

(18) $$K(s)=\frac{1.037\times 10^{13}s^{3}+4.788\times 10^{18}s^{2}+2.253\times 10^{19}s+3.661\times 10^{19}}{s^{4}+5.865\times 10^{5}s^{3}+5.901\times 10^{10}s^{2}+6.547\times 10^{14}s+2.498\times 10^{11}}.$$

**Figure 10:**
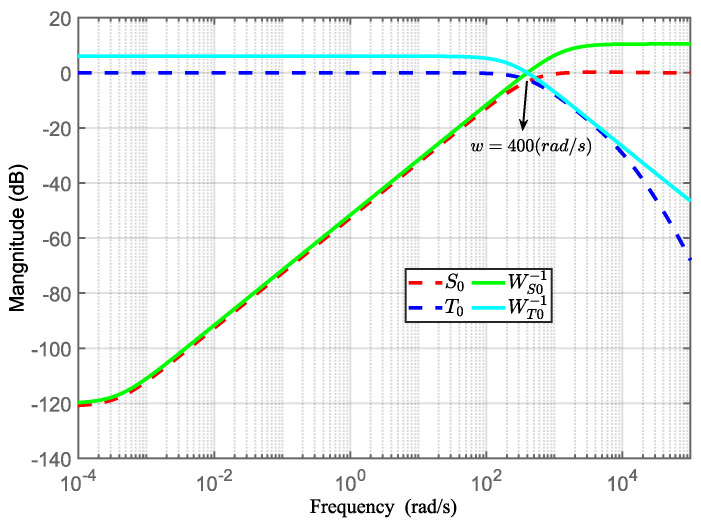
Frequency response of weighting function and sensitivity function.

Figure 10 shows the frequency response of the weighting function and sensitivity function. In the low-frequency region (−∞∼400 rad/s), the amplitude of *S*_0_(*s*) is small, which shows that *H*_∞_ control can eliminate the steady-state error in the system and suppress low-frequency disturbance to a certain extent. Similarly, in the high-frequency region (400 rad/s∼∞), the magnitude of *T*_0_(*s*) is also small to prevent the telescope pointing system from becoming sensitive to high-frequency noise.

**Figure 11:**
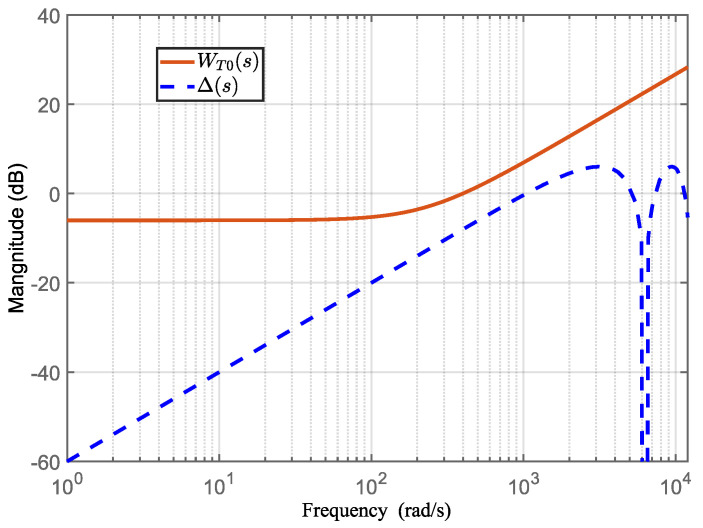
Robust stability analysis of the system.

The unmodeled part of the telescope pointing system contains a delay element (Δ(*s*) = *e*^−0.001*s*^ − 1). Figure 11 illustrates that the system uncertainty Δ(*s*) is enveloped by *W_T_*_0_(*s*)), indicating that the designed feedback controller ensures the stability of the telescope pointing system.

### 3.2. Optimized Design of Disturbance Observer

It can be seen in Figure 11 that in the low-frequency band, *W_T_*_0_(*s*) is not completely close to the system uncertainty Δ(*s*). This implies that the low-frequency disturbance suppression capability does not meet the ultra-high pointing stability requirements of the telescope pointing system. The reason for this lies in the presence of nonlinear friction; the *H*_∞_ controller cannot completely suppress the influence of nonlinear friction torque. To enhance the telescope pointing system’s ability to suppress nonlinear friction torque, a disturbance observer (DOB) was introduced to the inner loop of the control system. Additionally, to relax the constraints of the feedback controller and enhance the system’s disturbance rejection capability, the *H*_∞_ norm was utilized to optimize the filter *Q*(*s*) of the DOB system.

#### 3.2.1. Analysis of the Two-Degrees-of-Freedom System with DOB

**Figure 12:**
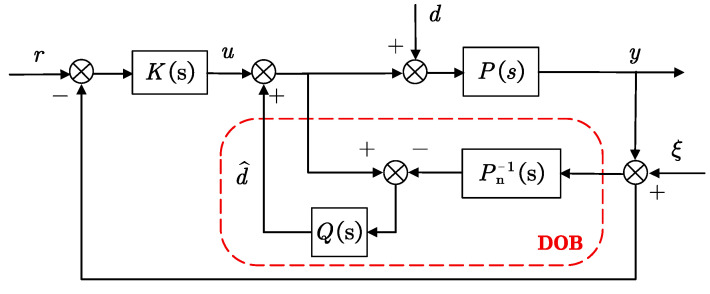
Feedback control system with DOB.

The feedback control system with the DOB system is shown in Figure 12, which consists of the DOB inner loop and the outer loop of the general feedback controller *K*(*s*). In the figure, *r*, *d*, *ξ,* and *y* are the reference input, disturbance, and DWS measurement noise and output signals, respectively.

The purpose of DOB is to eliminate the impact of external disturbance and model mismatch on the system. The low-pass filter *Q*(*s*) not only makes the inverse model physically realizable but also suppresses measurement noise and ensures the system’s robust stability to model mismatch. When matching the model (*P*_n_ = *P*), the output equation of the two-degree-of-freedom system is

(19) $$y=\frac{P_{n}(s)K(s)}{1+P_{n}(s)K(s)}r+\frac{P_{n}(s)(1-Q(s))}{1+P_{n}(s)K(s)}d+\frac{P_{n}(s)K(s)+Q(s)}{1+P_{n}(s)K(s)}\xi .$$

From Equation (19), it can be inferred that the influence of system disturbances and measurement noise on the system output is not only related to the filter *Q*(*s*) but also to the external feedback system of the system. The sensitivity function and complementary sensitivity function of a two-degrees-of-freedom system are defined as follows:

(20) $$\left\{\begin{array}{l} S(s)=\frac{1-Q(s)}{1+P_{n}(s)K(s)}=S_{0}(s)\cdot S_{\mathrm{DOB}}(s)\\ T(s)=\frac{P_{n}(s)K(s)+Q(s)}{1+P_{n}(s)K(s)}=T_{0}(s)+S_{0}(s)\cdot T_{\mathrm{DOB}}(s) \end{array}\right.,$$

In Equation (20), *S*_DOB_(*s*) = 1 − *Q*(*s*)) and *T*_DOB_(*s*) = *Q*(*s*) represent the sensitivity and complementary sensitivity functions of the inner-loop return DOB system, respectively.

#### 3.2.2. Evaluation Function Definition

1. Evaluation function for a two-degree-of-freedom system:

According to Equations (19) and (20), the impact of disturbance *d* on the output depends on the sensitivity function *S*(*s*), and the effect of sensor noise *ξ* on the output depends on the complementary sensitivity function *T*(*s*). Meanwhile, based on the small gain theorem (see Equation (14)), the robust stability conditions of the closed-loop system also depend on *T*(*s*). In a two-degrees-of-freedom control system, we define the performance function of the disturbance observer with order and structure constraints as [26]:

(21) $$\begin{array}{l} \max\gamma ,\underset{Q(s)}{\min}{\left\|\left[\begin{array}{c} \gamma W_{S}(s)S(s)\\ W_{T}(s)T(s) \end{array}\right]\right\|}_{\infty }=\\ \max\gamma ,\underset{Q(s)}{\min}{\left\|\left[\begin{array}{c} \gamma W_{S}(s){\left(1+P_{n}(s)K(s)\right)}^{-1}(1-Q(s))\\ W_{T}(s)(P_{n}(s)K(s)+Q(s)){\left(1+P_{n}(s)K(s)\right)}^{-1} \end{array}\right]\right\|}_{\infty }<1 \end{array}.$$

In the evaluation function of the two-degrees-of-freedom system, the transfer function has a complex form, especially concerning the complementary sensitivity function related to the robust stability conditions. Due to the robust stability conditions and structural constraints, this *H*_∞_ control problem cannot be directly solved through systematic methods. It is necessary to transform this problem into a form that can be systematically solved. The performance function of the two-degrees-of-freedom system can be converted into the performance function of the inner-loop DOB system for solving.

2. Evaluation function for the inner loop DOB system:

The inner-loop DOB system, as indicated by the red dashed box in Figure 12, has an input–output equation when the controlled object and the model are consistent (*P*_n_ = *P*):

(22) $$\begin{array}{l} y=\frac{P}{1+Q[P-P_{n}]P_{n}^{-1}}u-\frac{P(1-Q)}{1+Q[P(s)-P_{n}]P_{n}^{-1}}d-\frac{PQ}{P_{n}+Q[P-P_{n}]}\xi \\ \;\;\;=P(s)u-P(s)(1-Q(s))d-Q(s)\xi \end{array}.$$

In the DOB system, without considering the outer loop, disturbances *d* and detection noise *ξ* pass through the sensitivity function (*S*_DOB_(*s*) = 1 − *Q*(*s*)) and complementary sensitivity function (*T*_DOB_(*s*) = *Q*(*s*)) of the DOB system, respectively, affecting the system. To ensure that the system was not influenced by external disturbances and measurement noise, the terms involving *d* and ξ in the equation above had to be minimized. This involved making 1 − *Q*(*s*) and *Q*(*s*) sufficiently small; however, the sum of these two terms is always equal to 1, making it impossible for both to be minimized simultaneously. Therefore, a trade-off needed to be considered during the design. Similar to the *H*_∞_ control mixed sensitivity problem, a weighted function was used to address the frequency trade-off issue. The evaluation function for the DOB system is defined as follows:

(23) $$\max\gamma ,\underset{\begin{array}{c} Q(s)\in \Omega_{k}\\ Q(s)\in RH_{\infty } \end{array}}{\min}{\left\|\left[\begin{array}{c} \gamma W_{C}(s)(1-Q(s))\\ W_{Q}(s)Q(s) \end{array}\right]\right\|}_{\infty }<1.$$

The evaluation Function (23) involves complex norm conditions and constraints related to orders and relative orders, making it impractical to solve using standard *H*_∞_ control problem-solving methods. Reference [27] transformed the optimization problem with order constraints into a standard *H*_∞_ control problem. Thus, Equation (23) can be further transformed into an unconstrained optimization problem:

(24) $$\max\gamma ,\underset{\tilde{K}(s)}{\min}{\left\|\left[\begin{array}{c} \gamma W_{C}(s){\left(1+\tilde{P}(s)\tilde{K}(s)\right)}^{-1}\\ W_{Q}(s)\tilde{P}(s)\tilde{K}(s){\left(1+\tilde{P}(s)\tilde{K}(s)\right)}^{-1} \end{array}\right]\right\|}_{\infty }<1,$$

where $\tilde{P}$(*s*) and $\tilde{K}$(*s*)) represent the virtual plant and virtual controller of the open-loop system. This problem, without any order constraints and satisfying the assumptions of the standard problem, can be solved systematically using an optimization algorithm to obtain the optimal filter *Q*(*s*). This design approach not only meets all the order requirements of DOB’s filter *Q*(*s*), such as order conditions, relative order conditions, and internal model order conditions, but also ensures global optimality and convergence in the systematic solving process.

3. Transformation of evaluation functions:

In the performance functions of the two-degrees-of-freedom system (see Equation (21)) and the DOB system (see Equation (23)), there is a fundamental difference in terms of robust stability. However, in terms of form, it is possible to transform the performance function of the two-degrees-of-freedom system into that of the DOB system. The specific steps are as follows:

On one hand, the condition ${\left\|W_{T}(s)T(s)\right\|}_{\infty }<1$ in Equation (21) is a sufficient condition for the robust stability of the system. By the sensitivity function *S*(*s*) and the properties of the absolute value of complex numbers, we can derive the inequality:

(25) $$\left|Q(j\omega )\right|<\left|W_{T}^{-1}(j\omega )(1+L(j\omega ))\right|-\left|L(j\omega )\right|,\;\forall \omega ,$$

where *L*(*s*) = *P*_n_(*s*)*C*(*s*) is the open-loop transfer function of the feedback system at the nominal state, only if the filter *Q*(*s*) satisfies this inequality, the entire control system will be stable. In other words, Equation (25) is a sufficient condition for the robust stability of the closed-loop system. Choosing a stable weighting function *W_TD_*(*s*) that satisfies

(26) $$\left|W_{TD}^{-1}(j\omega )\right|<\left|W_{T}^{-1}(j\omega )(1+L(j\omega ))\right|-\left|L(j\omega )\right|≜E(\omega ),\;\forall \omega .$$

The sufficient condition for robust stability in Equation (25) can be reformulated as

(27) $$\;{\left\|W_{TD}(s)Q(s)\right\|}_{\infty }<1.$$

On the other hand, the term $max\;\gamma ,\underset{Q(s)}{\min}{\left\|\gamma \cdot W_{S}(s)S(s)\right\|}_{\infty }<1$ in Equation (21) represents the performance evaluation function for disturbance rejection. According to Equation (20) with *T*(*s*), the evaluation function can be expressed as:

(28) $$\max\gamma ,\underset{Q(s)}{\min}{\left\|\gamma \cdot W_{s}(s){\left(1+L(s)\right)}^{-1}(1-Q(s))\right\|}_{\infty }<1.$$

Choose a weight function *W_SD_*(*s*) that satisfies

(29) $$\left|W_{s}(j\omega ){\left(1+L(j\omega )\right)}^{-1}\right|\leq \left|W_{SD}(j\omega )\right|,\;\forall \omega .$$

Then, the sufficient condition of Formula (28) can be expressed as

(30) $$\max\gamma ,\underset{Q(s)}{\min}{\left\|\gamma \cdot W_{SD}(s)(1-Q(s))\right\|}_{\infty }<1.$$

According to Equations (27) and (30), the evaluation function of the two-degrees-of-freedom system can be expressed as

(31) $$\max\gamma ,\underset{Q(s)}{\min}{\left\|\left[\begin{array}{c} \gamma W_{SD}(s)(1-Q(s))\\ W_{TD}(s)Q(s) \end{array}\right]\right\|}_{\infty }<1.$$

Equation (31) is the same as the evaluation function for the DOB system (see Equation (23)). Therefore, the original design problem for the two-degrees-of-freedom system’s filter *Q*(s) (see Equation (21)) can be transformed into a standard design problem for the inner-loop DOB system. The optimal filter for Equation (31) can be obtained through the standard *H*_∞_ control framework (see Equation (24)).

#### 3.2.3. Filter Design

**Figure 13:**
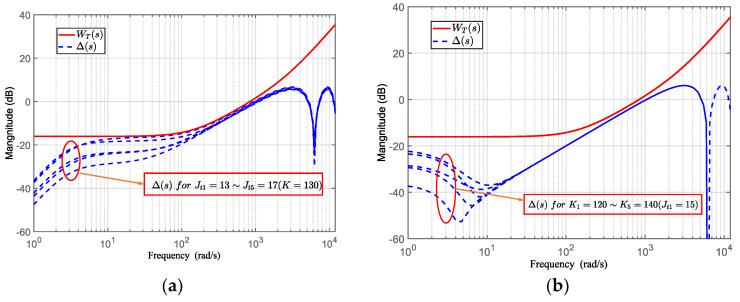
Robust stability analysis of the closed-loop system. (**a**) Perturbation model Δ(*s*) for the moment of inertia (*J_t_*_1_ = 13~*J_t_*_5_ = 17), with rotational stiffness held constant (*K* = 130). (**b**) Perturbation model Δ(*s*) for rotational stiffness (*K*_1_ = 120~*K*_5_ = 140), with moment of inertia held constant (*J_t_* = 15).

In the HODOB system, the choice of the weighting function in the optimal filter *Q*(*s*) is crucial. First, considering the model uncertainties, for the telescope pointing system, the unmodeled part includes the system delay and parameter variations in the dynamic model (rotational inertia and rotational stiffness). The curve in Figure 13 reflects the frequency and amplitude variations in model perturbations caused by uncertainties in the rotational inertia and rotational stiffness. Considering the possible parameter variations in Figure 13, the upper limit function *W_T_*(*s*) for uncertainties satisfying the frequency characteristic condition |Δ(*jω*)| < |*W_T_*(*jω*)| can be obtained. As shown in the figures, it is verified that the function

(32) $$W_{T}(s)=\frac{\left(s+140)(s+2800\right)}{2.5\times 10^{6}},$$

can be used as an upper limit function.

The choice of *W_TD_*(*s*)) should satisfy Equation (26), i.e., |$W_{TD}^{-1}$(*jω*)|<*E*(*ω*)| approaching *E*(ω) as closely as possible at high frequencies (beyond the cutoff frequency). In addition, the relative order of $W_{TD}^{-1}$(*s*) should be equal to the relative order of the open-loop system’s controlled virtual plant $\tilde{P}(s)$. According to the dynamic model of the telescope pointing mechanism in Section 2.3, the order of the controlled plant is 2. Considering the overall order *n* = 3 and the relative order *q* = 1 of the filter *Q*(*s*), the weighted function *W_TD_*(*s*) that satisfies Equation (26) can be chosen as:

(33) $$W_{TD}(s)=\frac{s^{2}+2700s+8.1\times 10^{4}}{2\times 10^{6}}.$$

**Figure 14:**
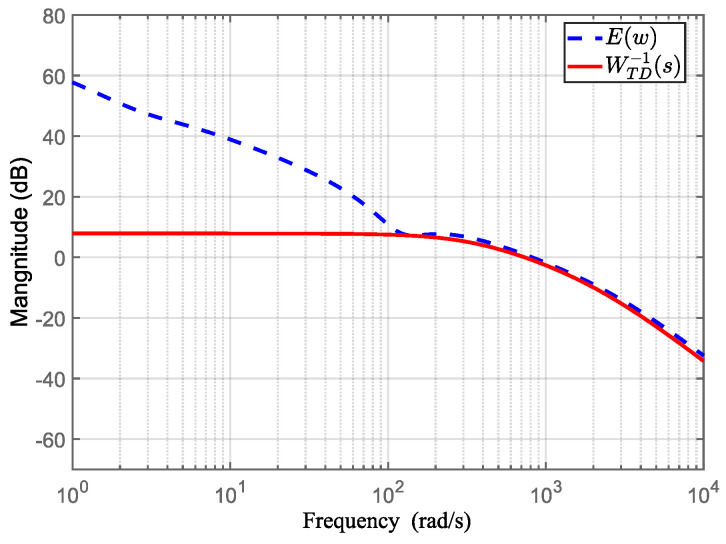
Frequency response of E(*ω*), $W_{TD}^{-1}$(s).

Figure 14 verifies Equation (26).

The selection of *W_SD_*(*s*) considers the overall order of *Q*(*s*) as *n* = *n*_w_ + *k* − 1, where n_w_ is the order of *W_SD_*(*s*) and k is the relative order of the controlled object. In the suboptimal solution, when maximizing γ to approach the optimal solution, the order is reduced by 1 [27]. Therefore, the weighting function *W_SD_*(*s*)) for low-frequency disturbance rejection performance can be chosen as:

(34) $$W_{SD}(s)=\frac{0.5{\left(s+1000\right)}^{2}}{10{\left(s+0.001\right)}^{2}}.$$

According to Equations (33) and (34), the optimal filter *Q* (*s*) can be obtained by using a robust control toolbox in Matlab (version R2021b) software

(35) $$Q(s)=\frac{1.9845\times 10^{6}s+4.316\times 10^{8}}{s^{3}+3129s^{2}+1.9845\times 10^{6}s+4.316\times 10^{8}}.$$

The above formula satisfies the order condition *n* = 3 and the relative order condition *k* = 2. For the general quadratic coefficient filter design [28], the form that satisfies the same conditions is

(36) $$Q_{b}(s)=\frac{3\sigma s+1}{{\left(\sigma s\right)}^{3}+3{\left(\sigma s\right)}^{2}+3(\sigma s)+1}$$

We chose *σ* = 0.005 and compared the low-frequency characteristics of this filter with the optimal filter from Equation (35) while ensuring consistency in the high-frequency characteristics.

**Figure 15:**
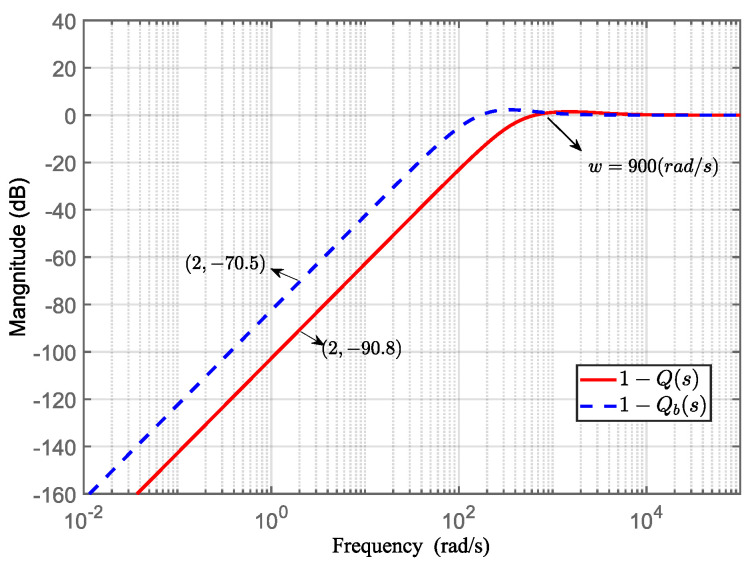
Comparison of disturbance suppression performance between optimally designed filter Q(*s*) and traditional filter design Q*_b_*(*s*).

Figure 15 presents a comparison of the low-frequency performance between 1 − *Q*(*s*) and 1 − *Q_b_*(*s*). In the frequency range below 900 rad/s, the magnitude of 1 − *Q*(*s*) is smaller than that of 1 − *Q_b_*(*s*). Specifically, in the frequency range below 200 rad/s, the amplitude difference is approximately 20 dB. This implies that under the same order condition, the disturbance suppression capability of the filter *Q*(*s*) optimized by the *H*_∞_ norm is 10 times higher than that of the traditional filter *Q_b_*(*s*).

## 4. Simulation and Analysis

This paper constructed a dynamic model of the telescope pointing mechanism in Matlab/Simulink (version R2021b) software, introduced disturbance noise into the model, and validated the effectiveness of the control method through numerical simulations. The reference signal in the simulation was derived from the respiratory angle variation in the TianQin constellation plane, using the calculated respiratory angle variation from orbital data as the ideal reference angle, as shown in Figure 2. The main parameters used in the simulation are listed in Table 1.

**Figure 16:**
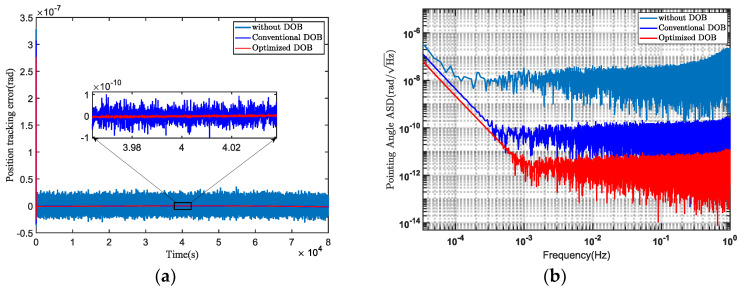
The control performances without DOB, with traditional DOB, and with optimized DOB are compared. (**a**) The tracking error. (**b**) The pointing stability.

In the numerical simulations, the tracking errors of the telescope pointing mechanism were compared under three scenarios: without DOB, traditional DOB design, and optimized DOB design, as shown in Figure 16a. In the graph, it can be observed that the tracking accuracy of the telescope pointing mechanism can be improved by approximately one order of magnitude using the HODOB method compared to the traditional DOB method. Converting the tracking errors into power spectral density, the simulation results in Figure 16b demonstrate that the use of HODOB significantly outperformed the traditional DOB in suppressing disturbance noise. This ensures that the stability of the telescope pointing system meets the pointing requirements of 10 nrad/Hz^1/2^ for the TianQin mission.

**Figure 17:**
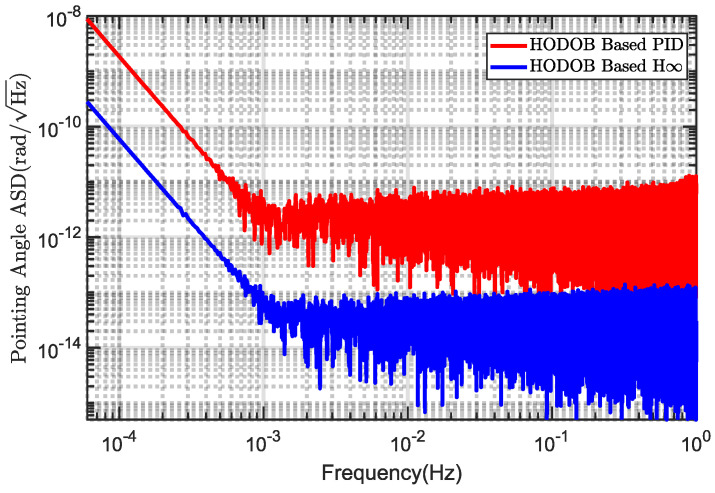
The pointing stability: Comparison of the control performances of HODOB-based *H_∞_* and HODOB-based PID.

To verify the effectiveness of the proposed composite control method, a comparison was made between the HODOB-based *H*_∞_ and the HODOB-based PID. Figure 17 illustrates the comparison results of the stability of the telescope pointing system under these two control methods. It can be observed in the figure that, within the detection frequency range, the proposed composite control method performed better in terms of pointing stability compared to the HODOB-based PID. In other words, the proposed composite controller exhibited greater robustness against multi-frequency band disturbances, thereby further enhancing the control performance of the telescope pointing system.

## 5. Conclusions

This paper presents a study of the high-precision pointing and tracking problem of a telescope pointing system subject to multiple disturbances. Addressing the model uncertainties and multiple disturbance challenges faced by the telescope pointing system, the study proposes a high-performance robust composite control approach, aiming to enhance the system’s robust stability and disturbance rejection capabilities. Firstly, the dynamic models for the telescope pointing mechanism and the actuator were established, and an analysis of the primary disturbance noise models affecting the telescope pointing system was conducted. Subsequently, considering the system’s uncertainties and torque disturbances, an *H*_∞_ mixed-sensitivity controller was designed. Additionally, to further attenuate and suppress nonlinear friction torque noise, an *H*_∞_ norm-optimized disturbance observer was introduced. The simulation analysis indicates that the HODOB method, compared to the traditional DOB design, improved the system’s tracking accuracy and pointing stability by an order of magnitude. Furthermore, the proposed composite control method enhanced the overall performance of the system, ensuring that the stability of the telescope pointing system far exceeds the requirements of the TianQin mission.

Therefore, utilizing this composite control approach enables the telescope pointing system to exhibit excellent control performance in terms of tracking accuracy, pointing stability, and robustness against multiple disturbances. This is of good reference value for the pointing control of the space gravitational wave detection’s respiration angle compensation. In our further works, we will conduct experimental research on the drive device of the telescope pointing system to validate the effectiveness of the control algorithms and improve the operational stability of the drive device.

## Figures and Tables

**Table 1:** Key simulation parameters.

Parameter	Value	Parameter	Value
J_t_	15 kg‧m^2^	($F_{\mathrm{Ej}}^{\mathrm{Oj}}$)_max_	5.7 × 10^−9^ N
J_s_	Diag{800,800,100} kg‧m^2^	($M_{\mathrm{Ej}}^{\mathrm{Oj}}$)_max_	3 × 10^−11^ Nm
ω_N_	2.9439 rad/s	$b_{j}^{S}$	3.6 × 10^−1^ m
ξ	0.9058	μ	398,600.44 km^3^/s^2^

## Data Availability

Data are contained within the article..
